# Migration of gastric cancer cells in response to lysophosphatidic acid is mediated by LPA receptor 2

**DOI:** 10.3892/ol.2013.1107

**Published:** 2013-01-07

**Authors:** DEZHI YANG, WENHUA YANG, QIAN ZHANG, YAN HU, LIANG BAO, ALATANGAOLE DAMIRIN

**Affiliations:** College of Life Sciences, Inner Mongolia University, Huhhot, Inner Mongolia 010021, P.R. China

**Keywords:** lysophosphatidic acid receptor2 (LPAR2), Gq/11, cell migration, gastric cancer

## Abstract

Lysophosphatidic acid (LPA), a natural phospholipid, is able to modulate diverse cellular responses through LPA receptors (LPARs). Several studies have reported that LPAR2 gene expression is increased in a variety of cancer cells, suggesting that LPAR2 is involved in gastric cancer. The present study investigated the expression profiles of the LPAR and involvement of the receptor subtypes in the LPA-induced migration of gastric cancer cells using cell migration assays, RNA interference, quantitative real-time PCR and western blotting. LPAR2 was observed to be highly expressed in SGC-7901 cells, a human gastric cancer cell line, while LPAR1 and LPAR3 were not. Transient transfection with LPAR2 siRNA was observed to reduce LPAR2 mRNA in SGC-7901 cells and eliminate the LPA-induced cell migration. It was also observed that LPA-induced SGC-7901 cell migration was inhibited by the inhibitor for Gq/11 protein and p38. The results suggest that the LPAR2/Gq/11/p38 pathway regulates LPA-induced SGC-7901 cell migration. The present findings suggest that LPAR2 may be a potential target for the clinical treatment of gastric cancer.

## Introduction

Tumors are associated with local bleeding which involves the activation of platelets during tumor development. Lysophospholipids are released from the activated platelets and subsequently converted to lysophosphatidic acid (LPA) by lysophospholipase (1). Therefore, LPA is considered to be highly expressed in tumors and regulate various tumorigenic processes, such as metastasis. LPA has been shown to induce diverse biological changes, including in Ca^2+^ mobilization, cAMP accumulation, cell shape, motility and proliferation in a variety of cell types (2–4). Extracellular LPA has also been observed to be involved in certain diseases (5–8) and have a positive role in the progression of ovarian, breast, colon and gastric cancer (9–11). These cellular responses to LPA are mediated by G protein-coupled receptors, i.e., several subtypes of LPA receptors (LPARs). At present, LPA1-6 receptors have been identified (3,4,12–17), among which LPA1–3 are members of the endothelial differentiation gene (Edg) family. LPA1–3 receptors have been investigated in the progression of gastric cancer (18,19). Immunohistochemical analysis of LPAR2 has shown that LPAR2 expression is a significant process in gastric cancer progression (20), although the mechanism of LPA-induced gastric cancer cell migration is not fully understood. The present study reports that LPA stimulates the migration of human gastric cancer cells (SGC-7901) and the LPAR2/Gq/11/p38 pathway regulates this migration.

## Materials and methods

### Cell culture and reagents

The human gastric cancer cell line SGC-7901 was provided by Institute of Zoology of China (Beijing, China). Human aortic smooth muscle cells (AoSMCs) were obtained from ATCC (Manassas, VA, USA). The cells were cultured in Dulbecco’s modified Eagle’s medium (DMEM; Gibco, Carlsbad, CA, USA) which was supplemented with 10% (v/v) fetal bovine serum (Gibco) at 37°C in a humidified atmosphere containing 5% CO_2_. 1-Oleoyl-sn-glycero-3-phosphate (LPA), fatty acid-free BSA and PTX were obtained from Sigma (St. Louis, MO, USA). The p-p38 and p38 antibodies were purchased from Santa Cruz Biotechnology (Santa Cruz, CA, USA) and Ki-16425 and YM-254890 were provided by Fumikazu Okajima (Gunma University, Maebashi, Japan) as gifts.

### Cell migration assays

Cell migration was measured using 24-well Transwell plates (Corning, Tewksbury, MA, USA), with 8 *μ*m-pore polycarbonate membranes. The Transwell plates were coated with 1% gelatin and the serum-free DMEM supplemented with LPA and 0.1% fatty acid-free BSA in the lower chamber was used as a lysophospholipid carrier. Cells (2×10^5^/ml) suspended in serum-free DMEM containing 0.1% fatty acid-free BSA were added to the upper chamber and incubated for 12 h at 37°C. When the effects of the LPA antagonists were examined, the cells were preincubated for 10 min with antagonists before being loaded. Unmigrated cells were removed from the top filter surface with a cotton swab and fixed with 100% methanol for 10 min. Migrated cells were observed to attach to the underside of the transwell plates and counted under a light microscope using a ×200 objective after stainning with 0.2% crystal violet. The experiments were repeated more than three times for each condition and for each experiment, five random fields were counted.

### RNA interference

Cells (3×10^5^) were incubated in a six-well plate overnight. Transient shRNA transfection was performed with Lipofectamine 2000 (Invitrogen, Carlsbad, CA, USA) according to the manufacturer’s instructions. Predesigned vectors expressing control shRNA- or LPAR2-specific shRNA were purchased from Inovogen (Inovogen, Beijing, China). The shRNA oligonucleotide sequence of LPAR2 was 5′-AGTACTTCCTACTGTTGGC-3′. The transfected cell clones were designated SGC-7901/shLPAR2 and SGC-7901/shRNA-control and the LPAR2 expression was detected by quantitative real-time PCR (RT-PCR) in these transfected cell clones.

### Quantitative RT-PCR

Total RNA was isolated with a total RNA isolation kit (Bio Basic Inc., Markham, ON, Canada) according to the manufacturer’s instructions. After DNase I (MBI Fermentas, Amherst, NY, USA) treatment to remove possible traces of genomic DNA in the RNA preparations, 5 *μ*g total RNA was used in reverse-transcription with the AMV First Strand cDNA Synthesis kit (Bio Basic Inc.). The primers used in the reaction were: LPAR1 forward, 5′-TCCTGTCCCGCGCCAGGTACAC-3′; LPAR1 reverse, 5′-GGTGGTGAACACGCCCCAGAACT-3′; LPAR2 forward, 5′-ACCGCAGTGTGATGGCCGTG-3′; LPAR2 reverse, 5′-TAGGAGCGGCTGAGCAGGGG-3′; LPAR3 forward, 5′-GCCGTGGAGAGGCACATGTC-3′; LPAR3 reverse, 5′-TGGCGATGGCCCAGACAAGC-3′; GAPDH forward, 5′-TCAAGTGGGGCGATGCTGGC-3′; GAPDH reverse, 5′-TGGGGGCATCAGCAGAGGGG-3′. Quantitative RT-PCR was performed using Hot Start Fluorescent PCR Core Reagent kits (Bio Basic Inc.). The cycling conditions were: 94°C for 4 min, then 35 cycles at 94°C for 30 sec and 60°C for 30 sec. The mRNA level of the genes of interest of each sample was normalized to that of the GAPDH mRNA and presented as unit values of 2^[Ct(GAPDH) - Ct(target gene)]^. Quantitative RT-PCR was performed in a Chromo4 detector (BioRad, Hercules, CA, USA).

### Western blotting

For the western blotting of p38, the cells were washed twice with ice-cold PBS and harvested from the dishes with a cell scraper by adding a WIP lysis buffer. The recovered lysate was incubated for 30 min on ice and centrifuged at 14,000 × g for 20 min to remove cell debris. The cell lysate was then subjected to gel electrophoresis for western blotting of the phosphorylated p38 and total p38.

### Statistical analysis

The Student’s t-test and one-way ANOVA using Graphpad Instat 5 software were used for the statistical analyses. P<0.05 was considered to indicate statistically significant differences.

## Results

### LPA-induces SGC-7901 cell migration is mediated by Gq-coupled receptor

**Figure 1:**
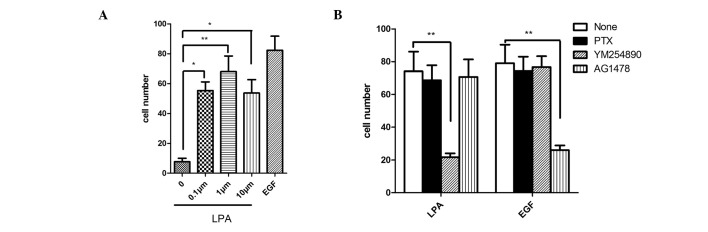
Involvement of Gq protein in LPA-induced SGC-7901 cell migration. (A) Migration activity of SGC-7901 cells was examined using transwell assays in the presence of LPA at 0.1, 1 and 10 *μ*M and EGF (20 ng/ml) as indicated. (B) SGC-7901 cells were pretreated with or without 50 ng/ml PTX for 16 h and treated with 1 *μ*M of YM-254890 or 1 *μ*M AG1487 for 1 h. The cells were further incubated with 1 *μ*M LPA or 20 ng/ml EGF to measure the cell migration. The values are the average (±SE) of six repeats from two separate experiments (^*^P<0.05, ^**^P<0.01). LPA, lysophosphatidic acid; EGF, epidermal growth factor; PTX, pertusis toxin.

LPA is a bioactive lysophospholipid that is known to induce diverse cellular responses by LPA G protein-coupled receptors (2). To detect the role of LPA in cell migration, SGC-7901 cells were stimulated with LPA at various concentrations (0.1, 1 and 10 *μ*M). LPA was observed to significantly increase cell migration and 1 *μ*M LPA was the most effective concentration (Fig. 1A). To identify which G protein is involved in LPA-induced SGC-7901 cell migration, YM-254890, a specific Gq protein inhibitor, was used in the cell migration experiment. It was observed that YM-254890 markedly reduced the LPA-induced cell migration. However, pertussis toxin (PTX) which inhibits Gi protein activity and AG1487, a specific inhibitor of epidermal growth factor (EGF) receptor, did not exhibit any effects on the LPA-induced cell migration. As shown in Fig. 1B, these G protein inhibitors did not affect EGF-induced cell migration, although AG1487 decreased the migration. The results indicate that Gq appears to be involved in LPA-induced cell migration but not by the EGF transactivation pathway (Fig. 1B).

### Antagonist for LPARs 1 and 3, Ki-16425, does not affect the migration of SGC-7901 cells

**Figure 2:**
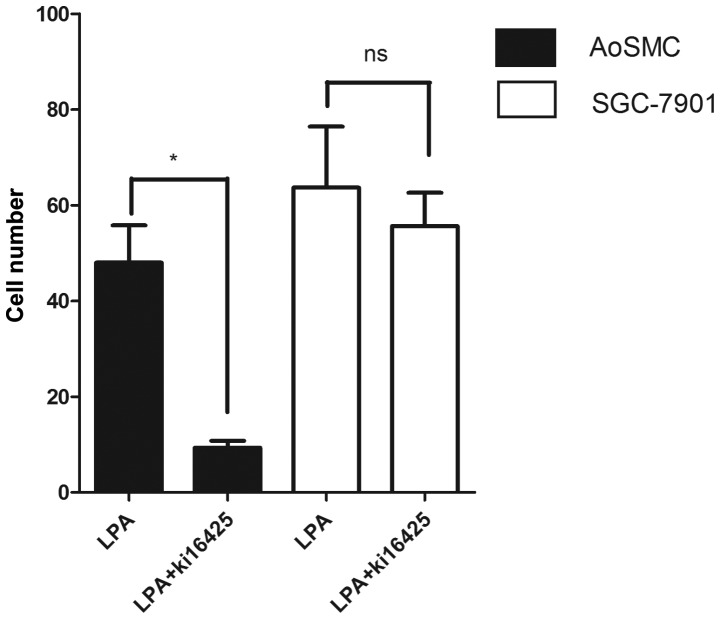
LPA-induced migration of AoSMC cells was inhibited by Ki-16425, but that of the SGC-7901 cells was not. The LPA-induced migration of SGC-7901 cells and AoSMCs was assayed after a 30-min incubation with or without 10 *μ*M Ki-16425, as indicated. The values are the average (±SE) of six repeats from three separate experiments (^*^P<0.05). LPA, lysophosphatidic acid; AoSMCs, aortic smooth muscle cells.

To understand the signaling pathways stimulated by LPA that lead to SGC-7901 cell migration, the role of Ki-16425 in cell migration was investigated. Ki-16425 is known to act as an antagonist of LPAR1 and LPAR3 (21). As shown in Fig. 2, the LPA-induced migration of the AoSMCs in which LPAR1 is highly expressed (22) was reduced to the level of the control in the presence of Ki-16425. However, Ki-16425 did not suppress the LPA-induced migration of SGC-7901 cells. These results suggest that the LPA-induced migration may not depend on LPAR1 and LPAR3.

### LPAR2 is highly expressed in SGC-7901 cells

**Figure 3:**
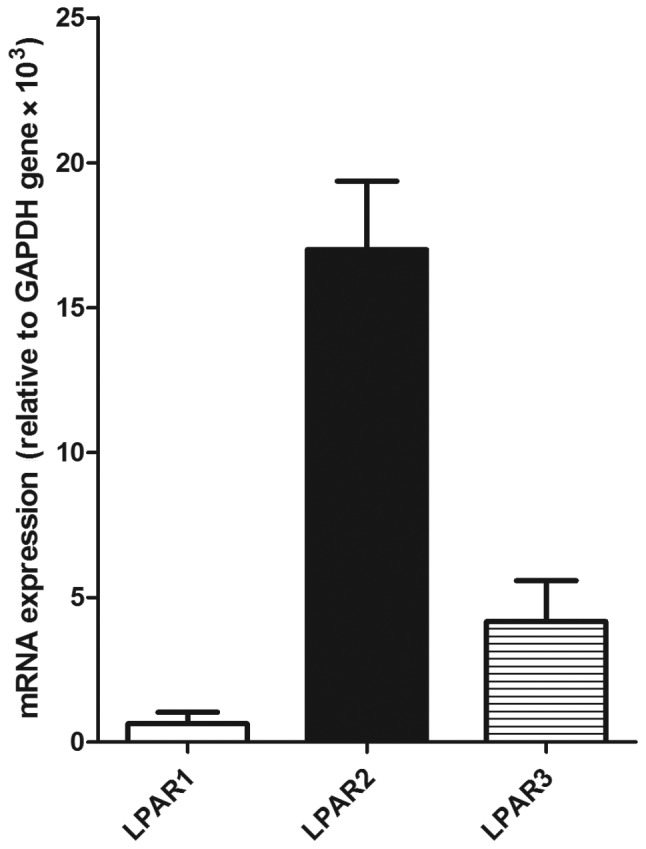
Expression of LPAR1-3 in SGC-7901 cells. The total RNA was isolated using a total RNA isolation kit. The mRNA level of LPAR1-3 was reverse transcribed, then assessed by real-time PCR. The values for expression are presented relative to GAPDH mRNA. The values are the average (±SE) of three repeats from a representative experiment. LPAR, lysophosphatidic acid receptor.

To evaluate the expression of LPARs 1-3 in the SGC-7901 cells, RT-PCR analysis was performed. LPAR2 was shown to be highly expressed in the SGC-7901 cells and it was 27- and 4-fold that of LPAR1 and LPAR3, respectively (Fig. 3). This result suggests that the LPA-induced migration of SGC-7901 cells may be dependent on LPAR2.

### Silencing LPAR2 expression by shRNA inhibits the LPA-induced cell migration of SGC-7901

**Figure 4:**
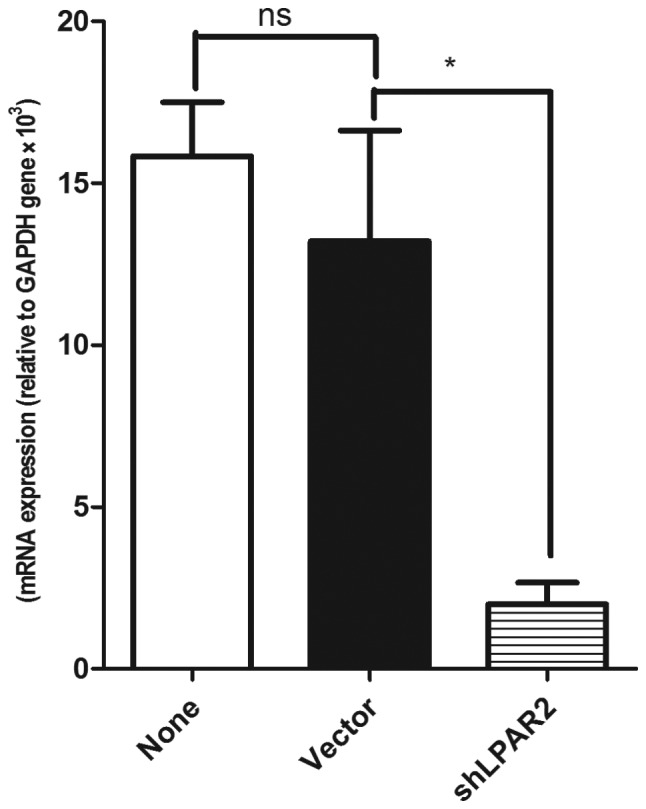
LPAR2 expression was silenced by specific shRNA in the SGC-7901 cells. Total RNA was isolated using a total RNA isolation kit. The mRNA level of LPAR1-3 was reverse-transcribed, then assessed by real-time PCR. The values are relative to GAPDH mRNA and are the average (±SE) of three repeats from a representative experiment (^*^P<0.05). LPAR2, lysophosphatidic acid receptor 2.

**Figure 5:**
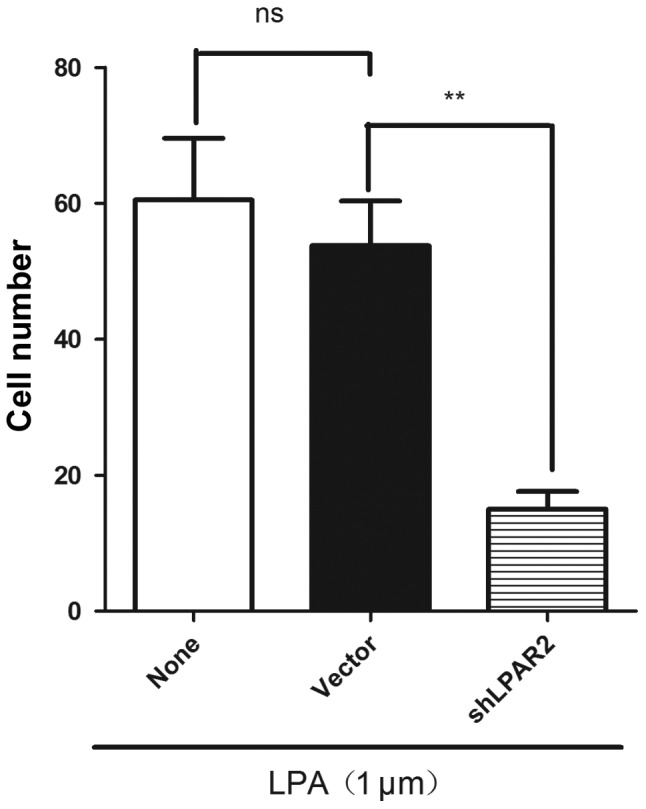
Silencing LPAR2 expression with shLPAR2 inhibits the LPA-induced cell migration of SGC-7901. LPA-induced SGC-7901 cell migration was assayed as described in the Materials and methods. The values are the average (±SE) of six repeats from three separate experiments (^**^P<0.01). LPAR2, lysophosphatidic acid receptor 2.

As mentioned, LPAR2 was highly expressed in the SGC-7901 cells, suggesting that LPAR2 may be important in LPA-induced cell migration. To investigate the role of LPAR2 in the LPA-induced cell migration, LPAR2 expression was silenced by LPAR2-specific shRNAs in the SGC-7901 cells. RT-PCR analysis showed that LPAR2 expression was decreased by 87.4% compared with the control (Fig. 4). Migration experiments showed that silencing LPAR2 expression significantly decreased the LPA-induced migration of SGC-7901 cells compared with the control SGC-7901 cells (Fig. 5). The results demonstrate that the LPA-induced migration of SGC-7901 gastric cancer cells requires LPAR2.

### Phosphorylation of p38 is required for the LPA-induced migration of SGC-7901 cells

**Figure 6:**
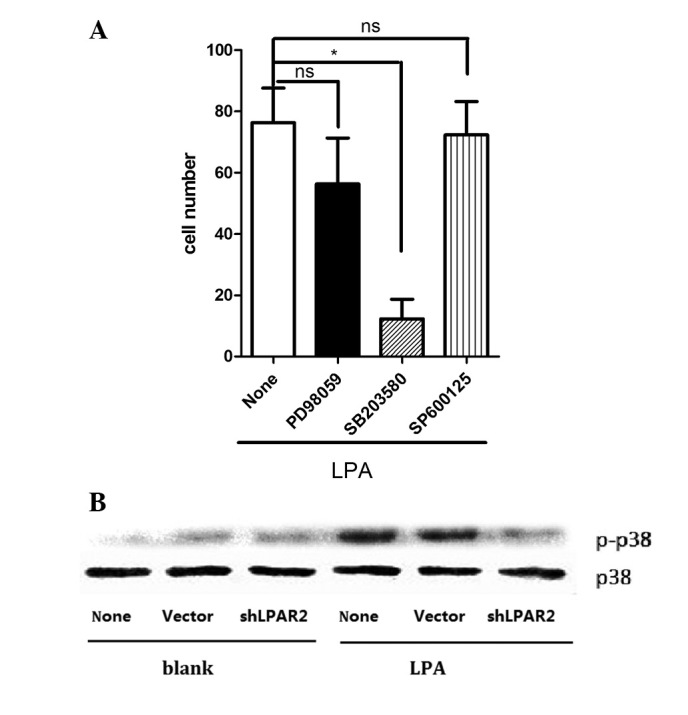
Phosphorylation of p38 is important for the LPA induced migration of SGC-7901 cells. (A) Effects of inhibitors on LPA-induced migration of SGC-7901 cells. The cells were pretreated with 10 *μ*M PD98059, 1 *μ*M SB203580 or 10 *μ*M SP600125 for 30 min at 37°C before stimulation with 1 *μ*M LPA. The migration experiment was performed as described in the Materials and methods. ^*^P<0.05. (B) LPA activates p38 MAPK phosphorylation. Non-transfected or transfected SGC-7901 cells with shLPAR2 or vector and treated with or without 1 *μ*M LPA were harvested and lysed. The cell lysates were subjected to western blot analysis using p38 and p-p38 antibodies. LPA, lysophosphatidic acid; LPAR, lysophosphatidic acid receptor.

To identify the mechanisms involved in LPA-induced cell migration, the effects of specific inhibitors for various kinases on cell migration were investigated. The presence of SB203580, a p38 MAPK inhibitor, was observed to significantly decrease the LPA-induced migration of SGC-7901 cells. However, neither PD98059 (an inhibitor of ERK kinase) or SP600125 (a JNK-MAPK inhibitor) affected LPA-induced cell migration (Fig. 6A). As shown in Fig. 6A, LPA activated the phosphorylation of p38 MAPK and the p38 MAPK phosphorylation was 4-5-fold that of control. The LPA-induced phosphorylation of p38 MAPK was attenuated by pre-transfection with shLPAR2 (Fig. 6B).

## Discussion

The enhanced migration observed in tumor cells is often caused by external stimuli and the sequential participation of cytoskeleton-related signaling molecules. However, the mechanism between the LPAR and G protein subtypes has not been analyzed in detail for LPA-induced migration in tumor cells. In the present study, the potential role of LPAR2 in gastric cancer SGC-7901 cell migration was evaluated. A previous study indicated that LPAR2 was correlated with a higher rate of lymphatic and venous invasion, lymphatic metastasis and the resulting tumor stage in diffuse-type gastric cancer (20). As chemotherapy supersedes radiation therapy as the standard therapeutic approach for advanced gastric cancer, the search for specific, effective and less toxic therapeutics becomes more critical (23). The present study identified LPAR2 as a potential new target. LPAR expression was previously unknown in SGC-7901 cells and the present study demonstrated that among the three principle LPARs, LPAR2 is predominantly expressed by SGC-7901 cells. The effect of LPA on gastric cancer migration with and without LPAR2 knockdown was then evaluated and the effect of LPA on migration was shown to be blocked in shLPAR2-transfected SGC-7901 cells. The inhibition of Gq by a specific Gq protein inhibitor also reduced SGC-7901 migration induced by LPA, indicating that Gq is responsible for LPA’s effect. Moreover, LPA induced p38 MAPK activation in SGC-7901 cells, while LPAR2 silencing reduced the effect of LPA.

The present study contributes to the use of specific shLPAR2 to identify the mechanisms and functions of LPAR2 and to investigate LPA’s regulatory effect on the gastric tumor microenvironment. The effects of LPA are mediated by the activation of the main three known LPARs and subsequent intracellular signal transduction. LPAR1, LPAR2 and LPAR3 belong to the endothelial differentiation gene family of G protein-coupled receptors (24). Through these receptors, LPA is implicated in numerous cellular processes, including cell proliferation and migration (25–27).

The LPARs’ individual signaling pathways have yet to be fully elucidated. LPAR2, in particular, has been shown to be upregulated in a variety of cancer types, including colon, gastric, ovary and endometrial cancer (20,28–30). LPAR2 is implicated in numerous oncogenic pathways and has been shown to transduce growth promoting signals in the LPA-rich environments characteristic of aggressive cancers (28). LPAR2 shares high homology in amino acid sequence with LPAR1 and LPAR3, with the exception of its carboxyl terminal region (31). This observation suggests that the cytoplasmic tails of the LPARs may specify their individual LPA signaling function. LPAR2 has been linked to specific receptor-interacting proteins such as TRIP-6, through which it induces ovarian cancer cell migration (31). LPAR2 has also been shown to increase LPA-mediated IL-6 and IL-8 production more efficiently than either LPAR1 or LPAR3 (32). Notably, the expression of the LPAR1 gene was observed to be significantly increased in atherosclerotic plaques in an atherosclerosis animal model. This finding demonstrates that the LPA-induced migration and proliferation of vascular smooth muscle cells are mediated by LPAR1 (unpublished study). However, LPAR2 has a similar role in gastric cancer cells, indicating that LPARs may have a cell specificity in their distribution and function.

In conclusion, LPAR2 is markedly expressed in SGC-7901 cells and, as a promising biomarker of gastric cancer, is critical in gastric cancer cell migration (invasion) in LPA-rich micro-environments. The pro-migratory effect of LPA is mediated by LPAR2 coupling to Gq and the p38 activation cascade in SGC-7901 cells. The present findings suggest that LPAR2 may be a potential target for the clinical treatment of gastric cancer. LPAR2 antagonists and inhibitors of its signaling pathway are potential drugs for this purpose.
